# Frequently reported adverse events of rebamipide compared to other drugs for peptic ulcer and gastroesophageal reflux disease

**DOI:** 10.1038/s41598-022-11505-0

**Published:** 2022-05-12

**Authors:** Eunkyeong Jang, Minju Park, Ji Eun Jeong, Ji Young Lee, Myeong Gyu Kim

**Affiliations:** 1 College of Pharmacy, Ewha Womans University, Seoul, 03760 Republic of Korea; 2 Graduate School of Pharmaceutical Sciences, Ewha Womans University, Seoul, 03760 Republic of Korea

**Keywords:** Health care, Medical research

## Abstract

This study aimed to detect safety signals of rebamipide and search for adverse events (AEs) of rebamipide that are more common than those of other drugs for peptic ulcer disease (PUD) and gastroesophageal reflux disease (GERD) in the elderly population. A total of 101,735 AE reports for drugs used to treat PUD and GERD between 2009 and 2018 from the KIDS-KAERS database (KIDS-KD) were used. Disproportionality analysis was performed to calculate the proportional reporting ratio (PRR), reporting odds ratio (ROR), and information component (IC). Drug labels in Korea, Japan, and China were reviewed to identify signals that have been listed. AEs frequently reported in the elderly population were also analyzed. Seriousness and median time to AEs were evaluated for statistically significant AEs. A total of 14 signals were detected, and 4 signals (dry mouth, dermatitis, purpura/petechia, and fluid overload) were not listed on drug labels; however, they may be included as part of other listed AEs. In the elderly population, 11 AEs such as dyspepsia/indigestion/gastrointestinal distress, somnolence, dry mouth, and edema were common. These AEs were not serious and occurred within 2–9 days. This study identified possible AEs of rebamipide, a relatively safe drug.

## Introduction

Rebamipide is a mucoprotective drug for peptic ulcer disease (PUD) and gastroesophageal reflux disease (GERD)^1^. It induces prostaglandins, resulting in increased blood flow to the gastric mucosa, mucous secretion, and enhanced mucosal defense. It also scavenges free radicals and inhibits inflammatory reactions^2^. In Korea, the size of the outpatient prescription market for rebamipide in 2020 was $90 million (approximately $0.08 a tablet). Rebamipide is known as a drug with few and mild adverse drug reactions (ADRs)^3^. The most common ADRs are gastrointestinal (GI) reactions such as nausea, vomiting, constipation, diarrhea, and bloating^4^.

Gastritis and GERD are the seventh and eighth most common diseases, respectively, in the Korean elderly population^5^. Proton pump inhibitors (PPIs) and histamine receptor antagonists (H2RAs) are representative drugs used to treat PUD and GERD. However, PPIs can increase the risk of *Clostridium difficile* infection, bone loss, and fractures in elderly patients; thus, it is recommended not to use PPIs for more than eight weeks^6^. In addition, H2RAs are generally avoided for patients with delirium even though the evidence for adverse cognitive effects is weak^7^. For this reason, rebamipide has been frequently used, which is considered relatively safe for the elderly. As the elderly population increases, it is necessary to determine whether there are any unknown or incompletely documented adverse events (AEs) of rebamipide by examining large-scale AE reports and verify the safety of rebamipide.

Pharmacovigilance is a scientific study and activity involving the detection, evaluation, interpretation, and prevention of drug-related problems^8^. Pharmacovigilance is crucial because it can identify a safety signal, defined as “reported information on a possible causal relationship between an AE and a drug, of which the relationship is unknown or incompletely documented previously”, and provide real-world evidence^9^. The Food and Drug Administration (FDA) Adverse Event Reporting System (FAERS) has become an important resource for pharmacovigilance analysis^10^. Similarly, the Korea Institute of Drug Safety and Risk Management (KIDS) developed the Korea Adverse Event Reporting System (KAERS) database (KIDS-KD). The KIDS-KD contains data for AEs that were spontaneously reported and can be used for pharmacovigilance analysis^10^. Renal neoplasm^11^ dementia^12^, acute kidney injury^13^, chronic kidney disease^13^, and hepatotoxicity^14^ have been evaluated for their causal relationship with PPIs in several pharmacovigilance studies. However, pharmacovigilance studies of rebamipide are limited. A study evaluated pulmonary AEs using the KIDS-KD^3,4^. Another study evaluated the efficacy of rebamipide in preventing non-steroidal anti-inflammatory drug (NSAID)-induced lower GI tract injury using the KIDS-KD and the Japanese Adverse Event Reporting Database^15^.

The aim of this study was first to detect safety signals of rebamipide that are not listed on drug labels using the KIDS-KD in general population, second to identify AEs of rebamipide that are more common than those of other drugs used for PUD and GERD in subgroup analysis of the elderly population.

## Results

### Characteristics of AE reports

**Figure 1:**
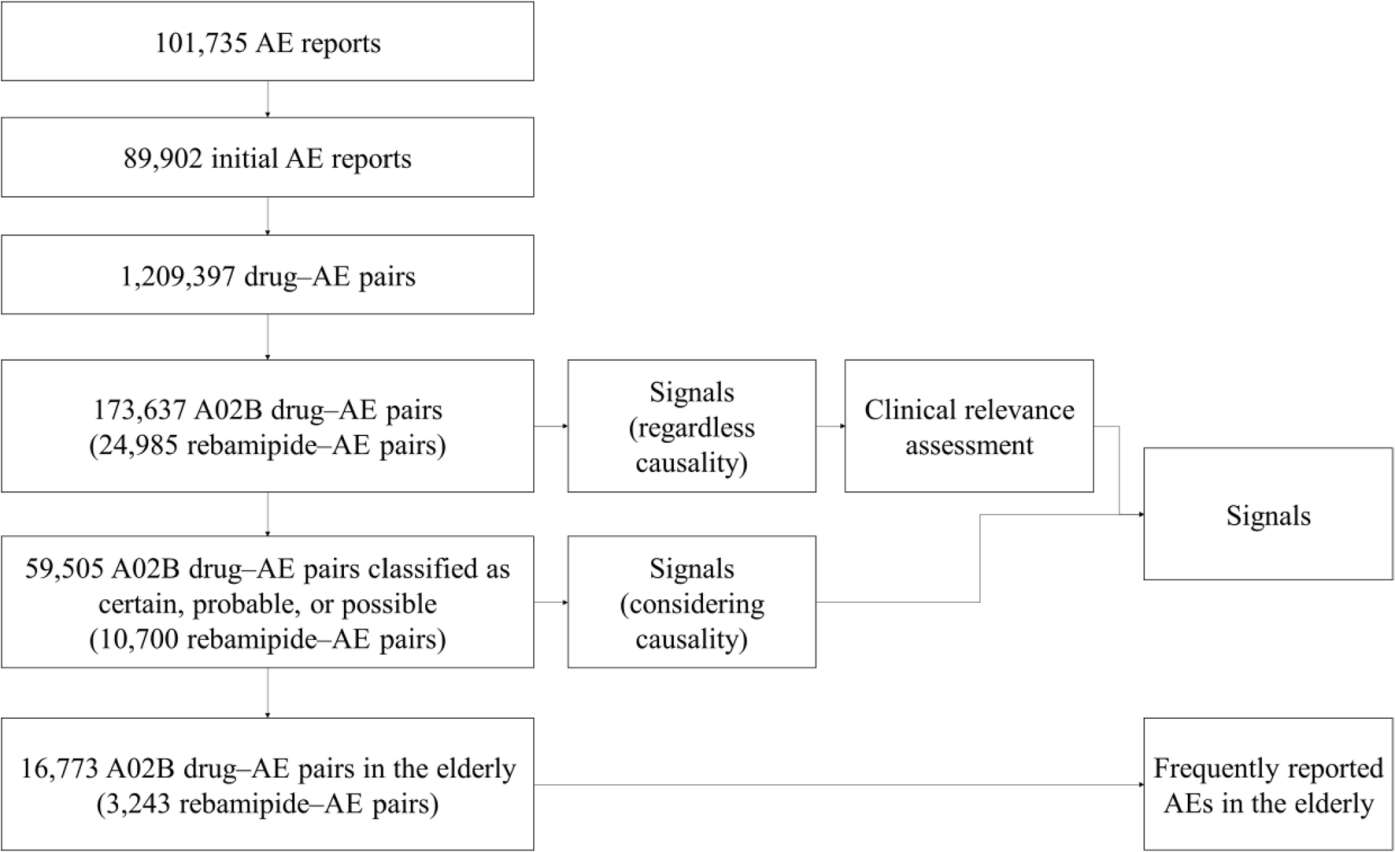
Study flow diagram.

Figure 1 shows the study flow diagram. There were 101,735 AE reports for drugs used to treat PUD and GERD between January 2009 and December 2018. We used 173,637 A02B drug–AE pairs for signal detection regardless of causality and 59,505 A02B drug–AE pairs classified as certain, probable, or possible for signal detection considering causality. Moreover, 16,773 A02B drug–AE pairs were used to determine frequently reported AEs in the elderly population. The characteristics of AE reports can be found in our previous study^3,4^.

### Signal detection

We detected 11 signals from drug–AE pairs classified as certain, probable, or possible. Table 1 shows these signals and their PRR, ROR, and IC values. Of the 11 signals, 8 signals were AEs listed on drug labels in three countries, which included somnolence, dyspepsia/indigestion/GI distress, face edema, generalized edema, malaise/feeling queasy, peripheral edema, periorbital edema, and thirst. As edema was included on the drug labels without further details, various types of edemas were evaluated as previously mentioned. Dry mouth, dermatitis, and purpura/petechia were not listed on the drug labels under the corresponding names.

**Table 1:** AE signals of rebamipide (analysis of drug–AE pairs classified as certain, probable, or possible).

AEs	Cases (rebamipide)	PRR	ROR	IC 95% CI	Label
Somnolence	1194	2.66	2.86	0.94	Yes
Dyspepsia/indigestion/GI distress	1115	2.13	2.26	0.73	Yes
Dry mouth	482	4.34	4.50	1.29	No
Face edema	247	2.78	2.82	0.86	Yes^†^
Generalized edema	166	3.44	3.48	1.00	Yes^†^
Malaise/feeling queasy	116	2.18	2.19	0.53	Yes
Peripheral edema	62	2.28	2.29	0.46	Yes^†^
Periorbital edema	54	2.07	2.08	0.33	Yes^†^
Thirst	43	5.16	5.18	1.02	Yes
Dermatitis	37	2.01	2.01	0.20	No
Purpura/petechia	31	2.14	2.15	0.21	No

*AE* adverse event, *CI* confidence interval, *GI* gastrointestinal, *IC* information component, *PRR* proportional reporting ratio, *ROR* reporting odds ratio.

^†^Labels including edema.

Regardless of causality, 15 rebamipide–AE pairs met the criteria of the PRR, ROR, and IC (Table 2). Of these AEs, 6 AEs were already listed on drug labels in Korea, Japan, and China. Among the other 9 AEs, dry mouth and fluid overload were clinically relevant.

**Table 2:** The AE signals of rebamipide (analysis of drug–AE pairs regardless of causality).

AEs	Cases (rebamipide)	PRR	ROR	IC 95% CI	Label	Clinical relevance
Somnolence	1285	2.94	3.04	1.11	Yes	–
Dry mouth	551	2.69	2.73	0.97	No	Yes
Generalized edema	198	2.03	2.04	0.58	Yes	–
Malaise/feeling queasy	144	2.20	2.20	0.63	Yes	–
Apathy	56	2.02	2.02	0.36	No	No^a^
Thirst	51	2.89	2.89	0.70	Yes	–
Vertigo	47	2.39	2.39	0.49	Yes	–
Micturition disorder	43	2.08	2.08	0.33	No	No^b^
Herpes simplex	25	2.12	2.13	0.18	No	No^c^
Pyelonephritis	22	2.30	2.30	0.21	No	No^d^
Increased stool frequency	15	2.23	2.23	0.01	Yes	–
Fluid overload	10	7.44	7.44	0.69	No	Yes
Aortic stenosis	10	3.30	3.31	0.10	No	No^e^
Vaginal pain	8	15.86	15.87	0.82	No	No^f^
Rheumatoid arthritis	6	5.10	5.10	0.04	No	No^g^

*AE* adverse event, *CI* confidence interval, *PRR* proportional reporting ratio, *ROR* reporting odds ratio.

^a^43 out of 56 (76.8%) used drugs that act on the central nervous system.

^b^35 out of 43 (81.4%) used anticholinergic agents.

^c^14 out of 25 (56.0%) used anticancer agents, and 6 out of 25 (24.0%) used immunosuppressive agents.

^d^4 out of 22 (18.2%) used antibiotics, 6 out of 22 (27.3%) used non-steroidal anti-inflammatory drugs (NSAIDs), 4 out of 22 (18.2%) used both antibiotics and NSAIDs, and 3 out of 22 (13.6%) used diuretics.

^e^All had more than two risk factors for aortic stenosis (diabetes, dyslipidemia, and hypertension).

^f^5 out of 8 (62.5%) used topical povidone-iodine, 1 out of 8 (12.5%) used estriol, and 1 out of 8 (12.5%) used a topical antifungal agent.

^g^4 out of 6 (66.7%) had a history of rheumatoid arthritis.

A total of 14 signals were identified according to the results presented in both tables: somnolence, dyspepsia/indigestion/GI distress, dry mouth, face edema, generalized edema, malaise/feeling queasy, peripheral edema, periorbital edema, thirst, dermatitis, purpura/petechia, vertigo, increased stool frequency, and fluid overload. Dry mouth, dermatitis, purpura/petechia, and fluid overload were not listed on the drug labels.

### AEs frequently reported in the elderly population

Table 3 shows 11 AEs that were common in the elderly population. Dyspepsia/indigestion/GI distress (ROR = 2.24), somnolence (ROR = 2.23), dry mouth (ROR = 3.97), and face edema (ROR = 3.04) were representative AEs reported substantially more than other A02B drugs. All AEs, except for purpura/petechia, were significant in subgroup analysis. There was no serious AE, and the median time to AEs ranged from 2 to 9 days.

**Table 3:** Frequently reported AEs in the elderly population.

AEs	Cases (rebamipide)	ROR (95% CI) vs. all A02Bs	ROR (95% CI) vs. H2RAs	ROR (95% CI) vs. PPIs	Median time to events
Dyspepsia/indigestion/GI distress	391	2.24 (2.00, 2.52)	2.49 (2.17, 2.85)	2.11 (1.83, 2.43)	4 days
Somnolence	274	2.23 (1.94, 2.56)	1.76 (1.51, 2.05)	2.78 (2.30, 3.35)	3 days
Dry mouth	209	3.97 (3.30, 4.76)	10.26 (7.52, 14.01)	2.06 (1.69, 2.52)	4 days
Face edema	90	3.04 (2.32, 3.97)	4.25 (3.00, 6.02)	2.02 (1.49, 2.75)	3 days
Abnormal temperature sensation /hot flashes	55	2.44 (1.75, 3.39)	2.12 (1.47, 3.06)	2.36 (1.56, 3.57)	2 days
Generalized edema	52	2.85 (2.01, 4.04)	5.57 (3.36, 9.23)	1.55 (1.06, 2.27)	6 days
Flatulence	41	2.15 (1.48, 3.13)	3.06 (1.91, 4.89)	1.66 (1.07, 2.57)	4 days
Malaise/feeling queasy	36	2.22 (1.49, 3.32)	2.06 (1.31, 3.24)	2.37 (1.42, 3.97)	4 days
Purpura/petechia	22	2.14 (1.29, 3.58)	2.58 (1.40, 4.76)	1.44 (0.81, 2.57)^†^	5 days
Thirst	18	4.71 (2.41, 9.23)	10.04 (3.40, 29.65)	2.58 (1.22, 5.46)	9 days
Periorbital edema	16	2.68 (1.43, 5.01)	2.54 (1.24, 5.21)	2.29 (1.07, 4.94)	3 days

*AE* adverse event, *CI* confidence interval, *GI* gastrointestinal, *H2RA* histamine 2 receptor antagonist, *PPI* proton pump inhibitor, *ROR* reporting odds ratio.

^†^Not significant.

## Discussion

We detected the safety signals of rebamipide in this study, which is the first to detect safety signals and compare the AEs of rebamipide with those of other drugs used to treat PUD and GERD in elderly patients.

A total of 14 signals were detected, and most were already included on drug labels. Signals not included on the drug labels (dry mouth, dermatitis, purpura/petechia, and fluid overload) may be included as part of other listed ADRs. Dry mouth is one of the peripheral signs and attributes of thirst, which is listed on drug labels^16^. Dermatitis is a general term that describes a common skin irritation and a group of drug hypersensitivity reactions involving the skin^17,18^. Hypersensitivity reactions such as urticaria, rash, itching, and eczema are known ADRs of rebamipide and can be regarded as dermatitis. Purpura and petechia can be associated with a decreased platelet count, which is indicated on drug labels^19^. Edema, which is listed on drug labels, can be regarded as fluid overload. Nevertheless, for clarity, these signals should be included on the drug labels.

Regardless of causality, some AEs met the criteria of signal detection; however, they may be explained by the patient’s underlying diseases or co-administered drugs. For example, apathy has been well described in patients with psychiatric diseases such as major neurocognitive disorders, schizophrenia, and major depressive disorder and taking drugs that act on the central nervous system (CNS), such as selective serotonin reuptake inhibitors^20^. Based on the results, 76.8% of apathy cases involved the use of drugs that act on the CNS. Another AE, micturition disorder, can be explained by co-administration with anticholinergic agents (81.4%). Anticholinergic agents are well-known drugs that cause voiding difficulties^21^. Herpes simplex virus (HSV) infection is common in patients receiving cytotoxic therapy for cancer or other immunosuppressive agents^22,23^. In this study, 56% of herpes simplex cases involved anticancer agents, and 24% of cases involved immunosuppressive agents. Pyelonephritis is nephritis due to ascending infection, and interstitial nephritis is nephritis caused by an allergic reaction to medication. Therefore, this information may have been reported incorrectly. Additionally, antibiotics, NSAIDs, and diuretics are the most common causes of interstitial nephritis^24^. In our study, most pyelonephritis cases involved the use of these causative agents. Risk factors for the development of aortic stenosis include hypertension, hyperlipidemia, and diabetes^25^. In cases of aortic stenosis in this study, all patients had more than two of these clinical risk factors. Vaginal symptoms may be attributed to irritants (e.g., povidone-iodine, soaps and perfumes, and some topical drugs) and allergens (e.g., latex condoms, topical antifungal agents, seminal fluid, and chemical preservatives) that elicit acute and chronic hypersensitivity reactions, including contact dermatitis. In our study, 62.5% of vaginal pain cases involved topical povidone-iodine, 12.5% of cases involved estriol, and 12.5% of cases involved a topical antifungal agent. Among patients with rheumatoid arthritis, 66% of them had a history of rheumatoid arthritis.

There were 11 common AEs in the elderly population. Although there were no serious AEs, AEs occurred in a short time and can be dangerous if the events overlap with those of other drugs. Polypharmacy is prevalent in elderly patients because of their underlying diseases. Particularly, somnolence, dry mouth, and edema are AEs that especially elderly patients should be cautious about considering that daytime sleepiness is associated with fracture risk^26^, dry mouth leads to trouble chewing, swallowing, tasting, or speaking, and edema can cause increasingly painful swelling, difficulty walking, decreased blood circulation, and an increased risk of infection in the swollen area. Some medications can worsen these symptoms when co-administered with rebamipide. For example, antidepressants, antipsychotics, antiepileptics, and opioids should be avoided for elderly patients because any combination of three or more of these CNS-active drugs increases the risk of falls and fracture^7^. The combination of rebamipide with antihistamines or anticholinergics needs to be monitored because these drugs can induce somnolence and dry mouth^27,28^. Moreover, antihypertensive agents, NSAIDs, steroids, estrogens, and certain diabetic medications known as thiazolidinediones can increase the risk of edema. Therefore, caution is needed when these drugs are used in combination with rebamipide for elderly patients. In these cases, other drugs for PUD and GERD, such as PPIs or H2RAs, may be safer options.

This study has some limitations. First, the KAERS collects only spontaneously reported AEs, and AEs can be underreported^29^. Underreporting can lower PRR or ROR values, resulting in fewer opportunities to detect signals that are statistically significant. Second, the quality of the KIDS-KD data is determined by the reporter. Reporters may misevaluate AEs, which may be attributed to underlying diseases and other drugs, or omit critical information.

We identified some frequently reported AEs of rebamipide compared to other drugs for PUD and GERD. Rebamipide is known as a relatively safe drug; however, the findings are meaningful as they demonstrate possible AEs of rebamipide. Further research should be conducted on the AEs of rebamipide identified in this study.

## Methods

### Data processing

We obtained spontaneous AE reports including drugs used to treat PUD and GERD between January 2009 and December 2018 from the KIDS. The study protocol was exempted from review by the institutional review board of Ewha Womans University (institutional review board number: ewha-202102-0009-01).

The data consisted of ASCII format tables: basic information (ADR_REPORT_BASIC), drug information (DRUG_INFO_ADR), AE information (ADR_INFO_REPORT), seriousness of AEs (SERIOUS_ADR), reporter information (REPORTOR_ADR), primary causality assessment (ASSESSMENT_ADR), and medical history (HIST_ADR).

AEs were coded according to the World Health Organization Adverse Reaction Terminology (WHO-ART) version 092, and drugs were coded using the Anatomical Therapeutic Chemical (ATC) classification system. Causality in the ‘ASSESSMENT_ADR’ table was judged by the reporter as ‘certain’, ‘probable’, ‘possible’, ‘unlikely’, ‘unclassified’, ‘unassessable’, or ‘not applicable’. As the reporter can report AEs without causality assessment, causality information can be omitted. Except for follow-up reports, only first reports were extracted, and drugs and AEs were paired.

### Disproportionality analysis

Disproportionality analysis is a method of detecting the AE signals of a specific drug. We constructed a 2 × 2 table, which has rows with rebamipide and all other drugs and columns with specific AEs and all other AEs (Table 4)^30^.

**Table 4:** 2 × 2 table for disproportionality analysis of rebamipide.

	Specific AEs	All other AEs
Rebamipide	A	B
All other drugs	C	D

(A) Rebamipide–specific AE pairs. (B) Rebamipide–all other AE pairs. (C) Other drug–specific AE pairs. (D) Other drug–all other AE pairs.

From the table, we calculated three signal indicators, i.e., proportional reporting ratio (PRR), reporting odds ratio (ROR), and information component (IC) (Table 5)^31^. All three indicators must meet the criteria to be signals of rebamipide. The lower limit of the 95% confidence interval (CI) of the IC was calculated according to a previous study^32^.

**Table 5:** Definition and criteria of signal detection for each indicator.

	Definition	Criteria of signal detection
PRR	(A/(A + B))/(C/(C + D))	PRR ≥ 2, chi-squared ≥ 4, and A ≥ 3
ROR	(A/B)/(C/D)	ROR ≥ 2, chi-squared ≥ 4, and A ≥ 3
IC	Log_2_(P(AE, drug)/P(AE) × P(drug))	Under limit of 95% confidence interval ≥ 0

*IC* information component, *PRR* proportional reporting ratio, *ROR* reporting odds ratio.

### Signal detection

**Figure 2:**
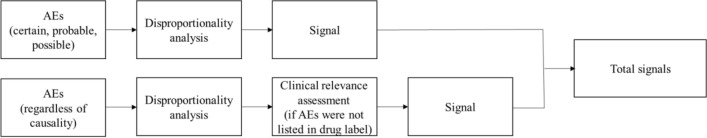
Process of signal detection.

Signal detection proceeded in two different ways (Fig. 2). In the first method, only drug–AE pairs classified as certain, probable, or possible from causality assessment were used in disproportionality analysis. In this case, AEs that met the criteria of the PRR, ROR, and IC were classified as signals.

However, rebamipide may not be considered as a causative drug because it is generally known as a relatively safe drug. In addition, causality assessment is not necessary for the KAERS, and some AE reports had no causality assessment. Therefore, in the second method, disproportionality analysis was performed regardless of causality information. AEs not included on the drug label were reviewed with co-administered drugs in the ‘DRUG_INFO_ADR’ table and medical history in the ‘HIST_ADR’ table to determine whether they were adverse effects caused by rebamipide. This process was conducted by an expert with more than 10 years of experience in clinical pharmacy.

AEs not caused by the action of drugs, such as prescription errors, were excluded. Drug labels in Korea, Japan, and China were reviewed to identify signals that have been listed. The ADR information listed on the labels of the three countries was the same.

### AEs frequently reported in the elderly population

Information on the patient’s age was recorded in ‘ADR_REPORT_BASIC’ table. Elderly population was defined as over 65 years of age at the time of AEs. Drug–AE pairs classified as certain, probable, or possible from causality assessment and reported among patients over 65 years of age were analyzed in this subgroup analysis. After constructing a 2 × 2 table, the ROR and 95% CI were calculated. We searched for AEs for which the lower limit of the 95% CI was greater than 1. The seriousness of frequent AEs was reviewed. Subgroup analysis was performed by limiting the control drugs to H2RAs (ATC A02BA) and PPIs (ATC A02BC). Additionally, we calculated the median time to AEs by subtracting the date of occurrence of AEs and the start date of taking rebamipide.

## Data Availability

KIDS-KD is available at the Korea Institute of Drug Safety & Risk Management (Ministry of Food and Drug Safety) website (https://open.drugsafe.or.kr/original/invitation.jsp). The authors do not have any special access privileges to this data. The datasets used and/or analyzed during this study are not publicly available due to privacy or ethical restrictions.
